# Meta-analysis with systematic review to synthesize associations between oral health related quality of life and anxiety and depression

**DOI:** 10.1038/s41405-024-00191-x

**Published:** 2024-02-13

**Authors:** Zainab Alimoradi, Elahe jafari, Zahra Roshandel, Marc N. Potenza, Chung-Ying Lin, Amir H. Pakpour

**Affiliations:** 1 Social Determinants of Health Research Center, Research Institute for Prevention of Non-Communicable Diseases, Qazvin University of Medical Sciences, Qazvin, Iran 3419759811,; 2 Department of Odontology and Oral Health Science, School of Health and Welfare, Jönköping University, Jönköping, Sweden; 3 Department of Psychiatry, Yale School of Medicine, 300 George St., New Haven, CT 06511 USA; 4 Institute of Allied Health Sciences, College of Medicine, National Cheng Kung University, Tainan, Taiwan; 5 University of Religions and Denominations, Qom, Iran; 6 Department of Nursing, School of Health and Welfare, Jönköping University, Jönköping, Sweden

**Keywords:** Oral-health-related quality of life, Oral hygiene

## Abstract

**Objectives:**

The present systematic review aimed to investigate how oral health related quality of life (OHQOL) associates with anxiety and depression. The study protocol was registered prospectively in the PROSPERO database (CRD42023389372).

**Materials and methods:**

Studies investigating associations between OHQOL and depression and/or anxiety were included. Fisher’s Z scores were used to summarize associations between OHQOL and depression/anxiety. Funnel plots and Begg’s Tests were used to assess publication bias. Meta-regression was conducted to examine potential moderator effects in the associations. Academic databases including the *ISI Web of Knowledge, Scopus, ProQuest* and *PubMed* were systematically searched. The quality of included studies was checked with the Newcastle Ottawa Scale (NOS).

**Results:**

All 15 included studies were cross-sectional (14,419 participants from nine countries; mean age=43.74 years). The pooled estimates showed weak associations between OHQOL and depression (Fisher’s z-score of 0.26 [95% CI = 0.17, 0.35; I^2^ = 96.2%; τ^2^ = 0.03]) and anxiety (Fisher’s z-score of 0.22 [95% CI = 0.001, 0.43; I^2^ = 97.9%; τ^2^ = 0.06]). No severe problems in methodology quality, publication biases, or moderator effects were observed.

**Conclusion:**

Both depression and anxiety were weakly associated with individuals’ OHQOL. Although the synthesized associations were not strong, they were significant, indicating that depression and anxiety are potential factors influencing individuals’ OHQOL.

## Introduction

As oral diseases represent health and economic burdens, promoting oral health (including oral health related quality of life (OHQOL)) is important [1, 2]. OHQOL may be impacted by oral conditions, diseases, and/or disorders [3]. Furthermore, OHQOL may impact general well-being [4]. Indeed, poor OHQOL has been with chronic health concerns, including but not limited to diabetes and cardiovascular diseases [5, 6].

Psychological health is important. However, current challenges to mental health are considerable. Prevalence estimates of anxiety and depression are considerable, especially after the COVID-19 pandemic, with depression prevalence estimated at 30.5% and anxiety at 25.0% [7]. OHQOL has been linked to depression and anxiety. Regarding potential etiologies, the individuals may not maintain good oral hygiene (e.g., tooth brushing) when depressed or anxious [8–12]. Depression may generate amotivation and interfere with performance of daily activities [13], including self-care behaviors and personal hygiene. Anxiety may generate worry that may disrupt daily routines [14, 15], leading to skipping of oral hygiene behaviors and resulting in poor or impaired oral health related quality of life. Alternatively, poor OHQOL may promote depression or anxiety [4, 16–18].

Although poor OHQOL may associate with depression and anxiety, empirical data are relatively limited and largely scattered, suggesting the need for integration of existing data. In this regard, how OHQOL relates to depression and anxiety may be synthesized using qualitative (i.e., a systematic review) and quantitative (i.e., meta-analysis) approaches. The synthesized qualitative and quantitative evidence could assist healthcare providers in designing appropriate programs to improve oral health related quality of life. Thus, we conducted a meta-analysis with a systematic review to provide insight into the presence and magnitudes of associations between OHQOL and depression and anxiety.

### Study aim

The present systematic review primarily aimed to investigate how OHQOL relates to anxiety and depression. Secondary objectives were to identify possible heterogeneity sources, moderators, and biases in publication.

## Materials and methods

### Design and registration

Following the present practice on systematic review with meta-analysis, the Preferred Reporting Items for Systematic Reviews and Meta-Analyses (PRISMA) guidelines were employed. Accordingly, the organization of the present findings followed the requirements proposed in the PRISMA guidelines [19]. Prospective registration of the study protocol was made in the PROSPERO (Decree code: CRD42023389372) [20].

### Criteria for eligible studies

The Population, Exposure, Comparison, Outcome, and Study design (PECO-S) components were used to identify potential articles for inclusion [21]. Specifically, eligibility criteria for included studies were:

- Populations included individuals at any age or of any gender/sex group;
- Exposure was depression and anxiety (assessed using valid and reliable measures);
- Comparison was not defined in the current study;
- Outcomes were the associations between OHQOL and depression and anxiety;
- Study design was defined as observational research using cross-sectional, cohort or case-control designs, published in English.

### Information sources

Information sources for the literature search included the *ISI Web of Knowledge, Scopus, ProQuest* and *PubMed*. The aforementioned databases were systematically searched from inception to April 2023. Additionally, manual searches of reference lists of the included studies were done to explore the gray literature.

### Search strategy

Based on PECO-S components [21], main search terms regarding the two main components of exposure (depression and anxiety) and outcome (oral-health-related quality of life) were selected. The sample search syntax for PubMed database was (((“quality of life”[tiab] OR “Life Quality”[tiab] OR “Health Related Quality Of Life”[tiab] OR HRQOL[tiab]) AND oral[tiab] AND health[tiab]) OR “oral health related quality of life”[tiab] OR “Oral Health Impact Profile”[tiab] OR “OHIP”[tiab] OR OHRQoL[tiab]) AND (Depression* OR (Depressive AND Symptom*) OR (Emotional AND Depression) OR Anxiet*). The search syntax was adopted for other databases based on their search features.

### Selection process

The selection process was conducted in two steps. First, titles and abstracts were checked and full texts of potentially relevant manuscripts were assessed based on the eligibility criteria. This process was conducted by two independent reviewers. In the event of disagreements during the selection process, the two reviewers convened to reach an agreed-upon decision.

### Data collection process and items

After selecting eligible papers, data collection was done by two reviewers independently using a pre-designed Excel spreadsheet. Data items included names of the first authors, publication dates, study designs, countries for data collection, numbers of participants, ranges and means of age, measures on assessment for OHQOL and depression/anxiety, countries’ income levels and development status according to world bank reports, and numerical results regarding associations between OHQOL and depression/anxiety. Of note, OHQOL was assessed using measures for which higher scores indicated worse oral-health-related quality of life. Any disagreements through the data collection process were resolved through discussion between the independent reviewers.

### Study risk of bias assessment

Methodological risk of bias in selection, comparability, and outcome assessment for included studies were examined using the Newcastle Ottawa Scale (NOS) for cross-sectional studies. Seven items rated the methodological quality. The maximum possible NOS score is nine. When overall scores are above five, studies are considered as having low risk of bias [22].

### Effect measure

Pearson’s correlation coefficients (and later converted to standardized Fisher’s Z scores showing effect sizes; please see *2.9 Synthesis methods* for details) were used as effect measures to present magnitudes of relationships between OHQOL and depression and anxiety. All forms of numerical findings (odds ratio, standardized mean differences) collected from included studies were transformed to Pearson’s correlation coefficients using approaches described on the psychometrica website (accessible at: https://www.psychometrica.de/effect_size.html).

### Synthesis methods

The quantitative synthesis in the meta-analysis was performed using the STATA software version 14. All analyses were performed, and the variance from within- and between-study was handled using the random effects models to resolve the issues of different populations derived from the included studies. Standardized Fishers’ z scores were computed to address potential instability of variance for Pearson’s *r*-correlation coefficients. Specifically, the following formula was used for the conversion: z = 0.5 × ln[(1+r)/(1-r)]) [23, 24] with SEz (i.e., standard error of z) =1/√ (n-3) [25]. The interpretation of the Fisher’s z is weak at 0.1; weak to moderate at 0.1 to 0.3; moderate at 0.3; moderate to strong at 0.3 to 0.5; and strong at 0.5 or above. Same as the Pearson correlation coefficient, negative values indicate inverse associations between examined variables. I^2^ index was used to check severity of heterogeneity [26].

### Reporting bias assessment

Publication bias was examined using Begg’s Tests and Funnel plots [27]. Publication bias was not assessed for the subgroup of studies investigating relationships between OHQOL and anxiety due to the low number of included studies (less than ten studies [28]). In order to rule out the probable single study effects on pooled effect sizes, sensitivity analysis was done, and the commonly used Jackknife method was applied [29].

### Moderator analyses

Meta-regression was done to assess potential effects of moderators in the associations between OHQOL and depression and anxiety. Via meta-regression, tau-square values (τ^2^ or Tau^2^) and adjusted R-squared and I-squared residuals were explored. In random-effects models, tau-squared values estimate between-study variance [30]. I-squared residuals reflect effects of selected variables on observed heterogeneity, with lower values reflecting greater heterogeneity [31]. Adjusted R-squared values indicate the proportion of between-study variance explained by covariates. Higher adjusted R-squared values indicate greater variance related to selected variables [32]. In meta-regression, consideration of the number of included studies when interpreting the significance of a *p*-value is important: a threshold of 0.20 can be used with less than 10 studies; a threshold of 0.15 can be used for 10 and 20 included studies; and a threshold of 0.10 can be used when there is above 29 studies [33, 34].

## Results

### Study screening and selection

**Fig. 1:**
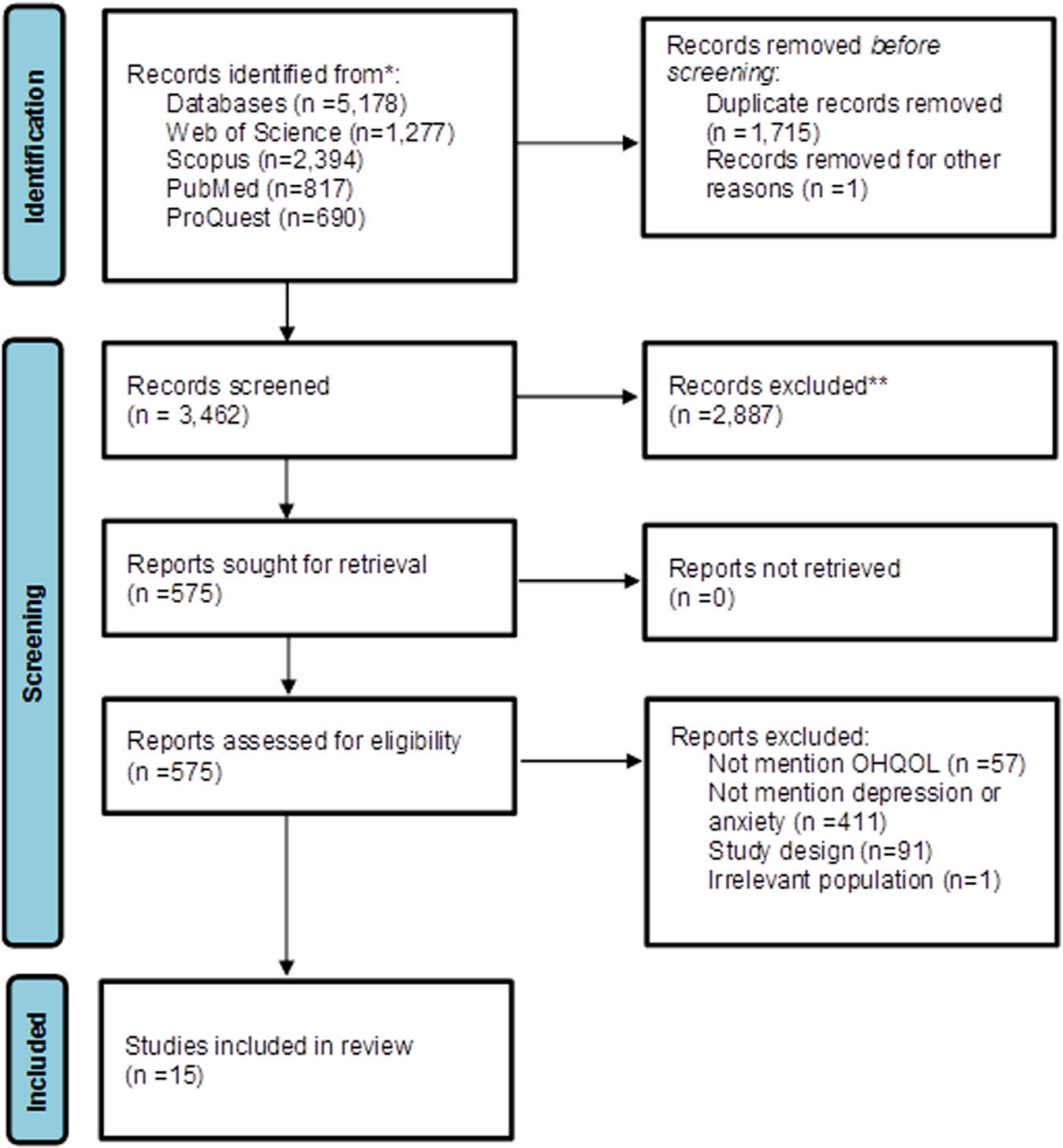
Search process based on the PRISMA flowchart. PRISMA flowchart showing the search, selection and analysis process.

Overall, 5,178 papers were retrieved after the systematic search was conducted in the *Web of Science* (*n* = 1277), *Scopus* (*n* = 2394), *PubMed* (*n* = 817) and *ProQuest* (*n* = 690) databases. After removing duplicates (*n* = 1716), the titles and abstracts of the remaining papers were screened. Finally, 15 studies were included. The PRISMA flowchart (Fig. 1) shows the search, selection and analysis process.

### Study description

Fifteen cross-sectional studies together having 14,419 participants from nine countries (Saudi Arabia, Romania, Korea, Japan, India, Germany, China, Brazil, and Australia) were included. The smallest sample size was 87 (from the Romania), and the largest sample size was 3615 (from Korea). The mean age of participants was 43.74 years with ages ranging between 15.9 and 77.9 years. Nearly half of the studies (7 out of 15) were conducted in high-income/developed countries. Overall, 54.92% of participants were female. Associations between OHQOL and depression were reported in 15 studies, while associations between OHQOL and anxiety were reported in five studies. Table 1 provides the summarized characteristics of included studies. Some studies reported crude data (5 out of 15), and some reported adjusted findings (10 out of 15). Due to the overall low number of included studies, we decided to include both crude and adjusted data and report them in Table 1. Also, one variable was defined as being adjusted or not which was included as a covariate in meta-regression, with results reported in Table 2.

**Table 1:** Summarized characteristics of included studies.

Study ID	Author	Country Income level Development status	Target Population and their Health Status	Sample size Age (years), mean Female %	Depression/ Anxiety measure	Oral Health QoL measure	NOS Total	variables adjusted
43	Kumar N et al., 2018 [35]	India Lower intermediate Developing	Older adults; Healthy	135 NR 60	PHQ-9	Geriatric OHAI	6	Not adjusted
47	Park K E et al., 2023 [36]	Korea High Developed	Older adults; Healthy	3615 72.5 57.1	CES-D10	Geriatric OHAI	6	age, sex, education level, spouse, living family members, health insurance type, household income, self-rated health, chronic disease, ADL
7	Hajek A et al., 2022 [37]	Germany High Developed	General population; Healthy	3075 44.5 51.1	PHQ-9 GAD-7	OHIP-G5	6	sex, age, family status, educational level, occupational status, smoking status, alcohol intake, sports activities,
16	Sebastiani AM et al., 2020 [38]	Brazil Upper intermediate Developing	Individuals with dentofacial deformity; Patients	132 30 75	axis II of the RDC-TMD	OHIP_14	7	Sex, Myofascial pain, Joint inflammation
1	Baldiotti ALP et al., 2023[39]	Brazil Upper intermediate Developing	Adolescents; Healthy	90 15.9 51.7	DC/TMD	OHIP_14	6	Subjective Happiness Scale, COMT Polymorphism rs174675 recessive
37	Thirunavukkarasu A et al, 2023 [40]	Saudi Arabia High Developed	T2 DM; Patients	677 46.7 47.1	DASS-21	OHIP_14	7	Not adjusted
15	Abdelsalam S et al., 2021[41]	Australia High Developed	People who inject drugs; Patients	943 36.5 34	PHQ-9	OHIP_14	6	dental service variables
12	Zhang Z et al., 2019- females [42]	China Upper intermediate Developing	General population; Healthy	2474 18.3 100	SDS	OHIP_14	6	age, ethnicity, sibling number, and parental education level, smoking status, drinking status, frequency of breakfast, and
6	Li H et al., 2022 [43]	China Upper intermediate Developing	Older adults; Healthy	613 66.3 73.1	DASS-21	General OHAI-12	7	Educational level, Migration years, Migration type, Hypertension, Gastrointestinal disease, Outpatient service attendance
12	Zhang Z et al., 2019- Males [42]	China Upper intermediate Developing	Students; Healthy	987 18.5 0	SDS	OHIP_14	6	age, ethnicity, sibling number, and parental education level, smoking status, drinking status, frequency of breakfast, and
9	Oancea R et al., 2020, [44]	Romania High Developed	Students; Healthy	87 24 58.2	PHQ-9	OHIP-49	3	Not adjusted
27	Noguchi S et al., 2016 [45]	Japan High Developed	Older adults; Patients	187 77.9 53.5	GHQ-12	Geriatric OHAI	6	Not adjusted
36	Satishkumar CS C et al., 2021[46]	India Lower intermediate Developing	Edentulous individuals; Patients	207 60.5 45.9	BDI	OHIP-EDENT	7	age, material status, education
26	Ohi T et al., 2021[47]	Japan High Developed	Older adults; Healthy	236 67.8 38	SDS	OIDP	6	age, sex, body mass index, hypertension, cerebrovascular/
41	Yap AU et al., 2021 [48]	China Upper intermediate Developing	People with differing TMD severity; patients	961 33 79.2	DASS-21	OHIP-TMD	7	Not adjusted

**Table 2:** Results of moderator analysis in associations between OHQOL and depression/ anxiety based on univariate regression.

Mental health problems	Variables	No of Studies	Coefficient	S.E.	*p*	I^2^ Res (%)	Adj. R^2^ (%)	Tau^2^
Depression (*n* = 15)	Study year	14	−0.01	0.01	0.35	93.36	1.36	0.02
	Country income level	15	−0.01	0.06	0.82	93.92	−6.44	0.02
	Country development status	15	−0.05	0.08	0.54	93.10	−1.90	0.02
	Target population	15	0.02	0.01	0.12	93.73	15.25	0.02
	Participants’ health status	15	−0.11	0.08	0.21	94.86	8.11	0.02
	Participants mean age (years)	14	−0.001	0.002	0.81	95.20	−7.02	0.02
	Percentage of female participants	15	−0.0001	0.002	0.96	96.35	−9.08	0.02
	OHQ measure	15	0.014	0.02	0.42	96.40	−2.71	0.02
	NOS total score	15	0.01	0.05	0.74	95.67	−4.87	0.02
	Using Adjusted data vs. crude data	15	−0.07	0.09	0.44	95.26	−0.86	0.02
Anxiety (*n* = 5)	Study year	4	0.06	0.06	0.45	90.67	−4.10	0.009
	Country income level	5	−0.17	0.20	0.45	95.08	−1.80	0.04
	Country development status	5	−0.17	0.20	0.45	95.08	−1.80	0.04
	Target population	5	0.04	0.02	0.18	95.66	35.68	0.03
	Participants’ health status	5	−0.26	0.17	0.21	95.78	28.22	0.03
	Participants mean age (years)	5	0.001	0.007	0.89	98.29	−32.72	0.06
	Percentage of female participants	5	0.01	0.005	0.09	92.81	58.07	0.02
	OHQ measure	5	0.06	0.02	0.09	95.95	58.60	0.02
	NOS total score	5	0.29	0.16	0.16	95.20	40.12	0.03
	Using Adjusted data vs. crude data	5	−0.26	0.17	0.21	95.78	28.22	0.03

### Quality assessment

All but one study (14 out of 15) were identified as having low risk of bias. The methodological quality of these studies is reported in Table 1 using the total scores. According to the methodological quality assessment, the following results were obtained:

i. Almost all studies (14 of 15) had selected participants which were representative or somewhat representative of the average in the target population.
ii. Sample size was not justified in most studies (10 of 15).
iii. None of the studies described the characteristics or the response rate among the non-responders and the responders.
iv. All studies assessed the exposure (depression and anxiety) using validated measurement tools.
v. Almost all studies (14 of 15) controlled for confounding factors based on the study design or analysis.
vi. All studies assessed the outcomes using self-report measures.
vii. Almost all studies (14 of 15) used clearly described statistical tests to analyze the data.

The two main methodological problems of the included studies were (i) not justifying the sample size and (ii) not providing a description regarding non-respondents.

### Outcome measures

#### OHQOL and depression

**Fig. 2:**
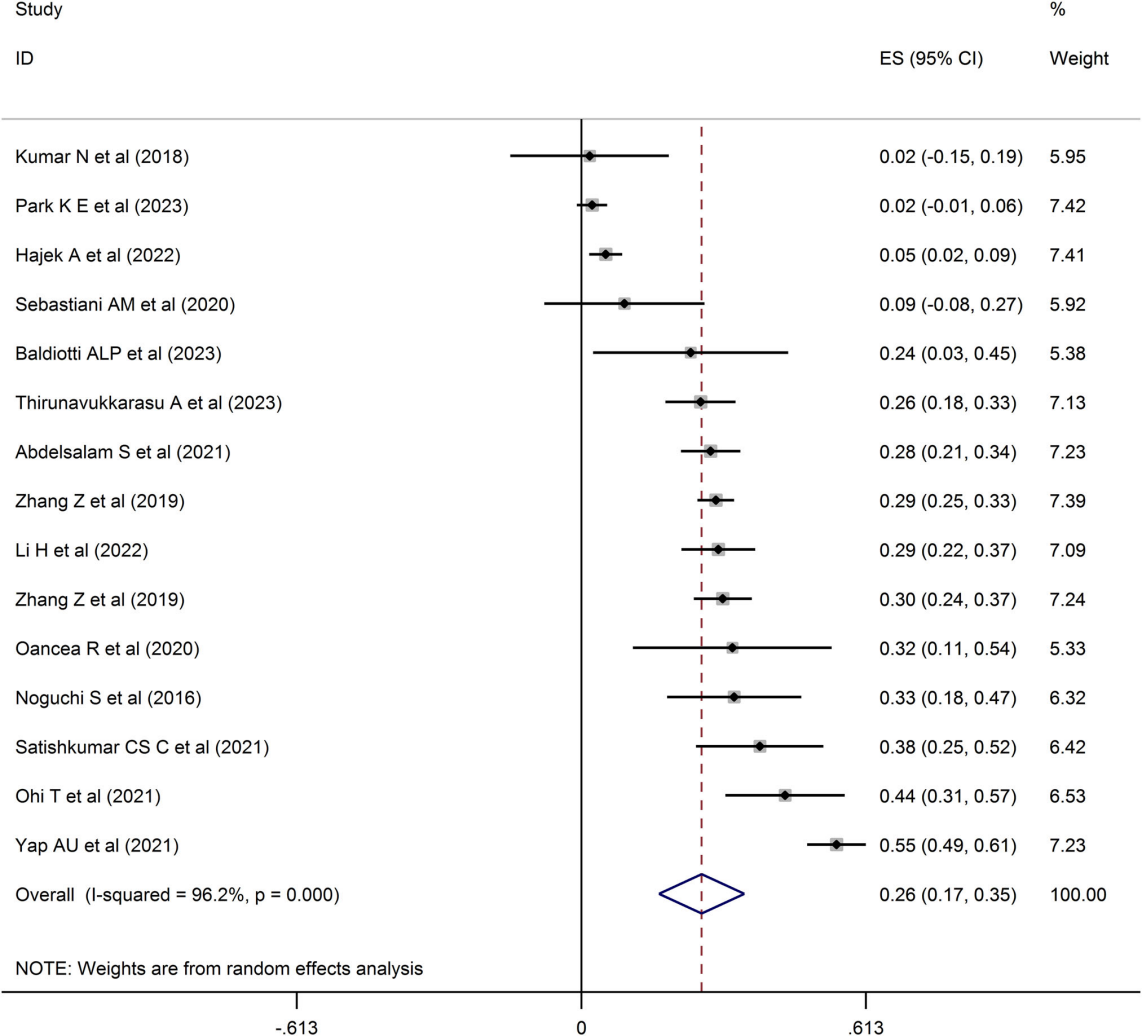
Forest plot displaying the estimated pooled Fishers’ z-score in the association between OHQOL and depression.

**Fig. 3:**
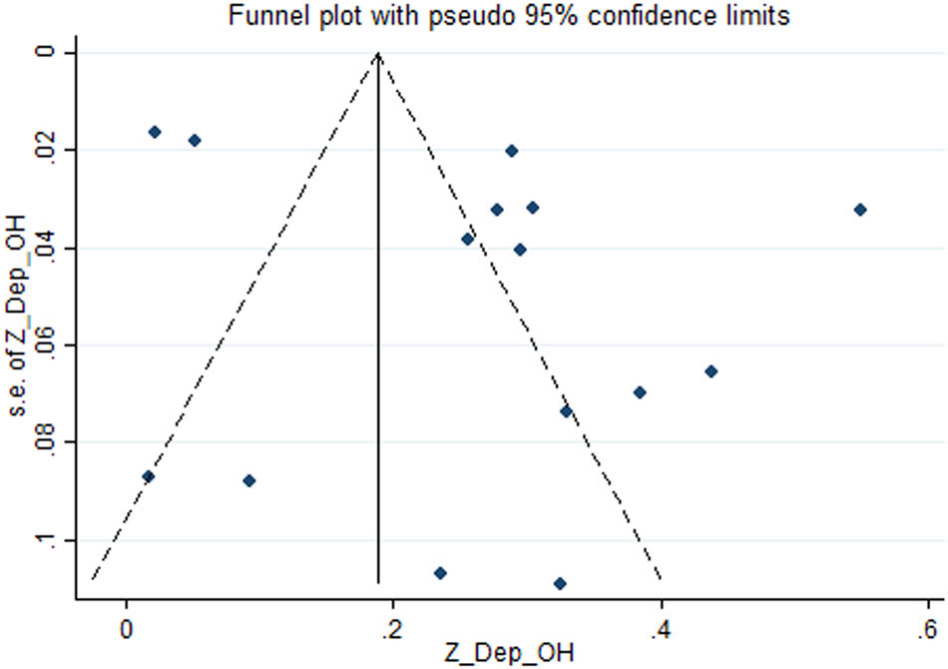
Funnel plot displaying the estimated pooled Fishers’ z-score in the association between OHQOL and depression.

**Fig. 4:**
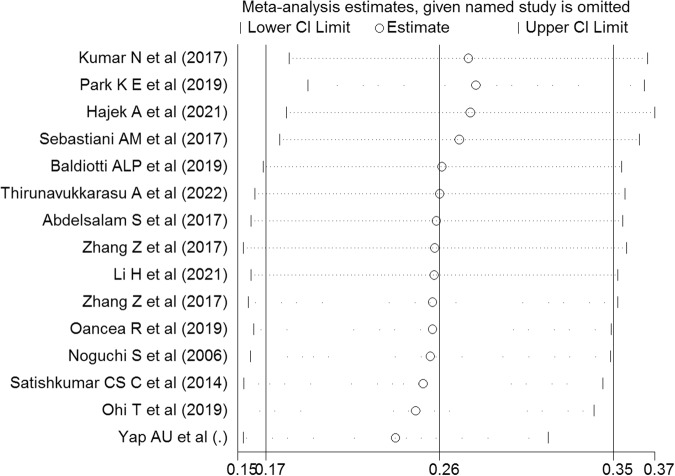
Sensitivity analysis plot assessing the small study effect in the estimated pooled Fishers’ z-score in the association between OHQOL and depression.

A weak association between OHQOL and depression was suggested by the pooled estimation. Specifically, the Fisher’s z-score was 0.26 with a 95% confidence interval (CI) ranging between 0.17 and 0.35 (I^2^ = 96.2%; τ^2^ = 0.03). The forest plot related to the association between OHQOL and depression is shown in Fig. 2. Publication bias in associations between OHQOL and depression was assessed based on the funnel plot (Fig. 3) with the Egger’s test (*p* = 0.11). The funnel plot seemed asymmetric, so further assessment was conducted using the fill-and-trim method. Seven studies were imputed in the fill-and-trim method to correct for probable publication bias. The corrected association between OHQOL and depression was 0.14 using the value of Fisher’s z-score (95% CI: 0.05–0.23, τ^2^ = 0.04). Additionally, the sensitivity analysis indicated that no single study impacted the pooled effect size (Fig. 4). Via the findings from uni-variable meta-regression, heterogeneity in the association between OHQOL and depression was not impacted by any examined variables, including the OHQOL measures. The only potentially significant moderator in this association was the target population (explaining 15.25% of the variance, *p* = 0.12).

#### OHQOL and anxiety

**Fig. 5:**
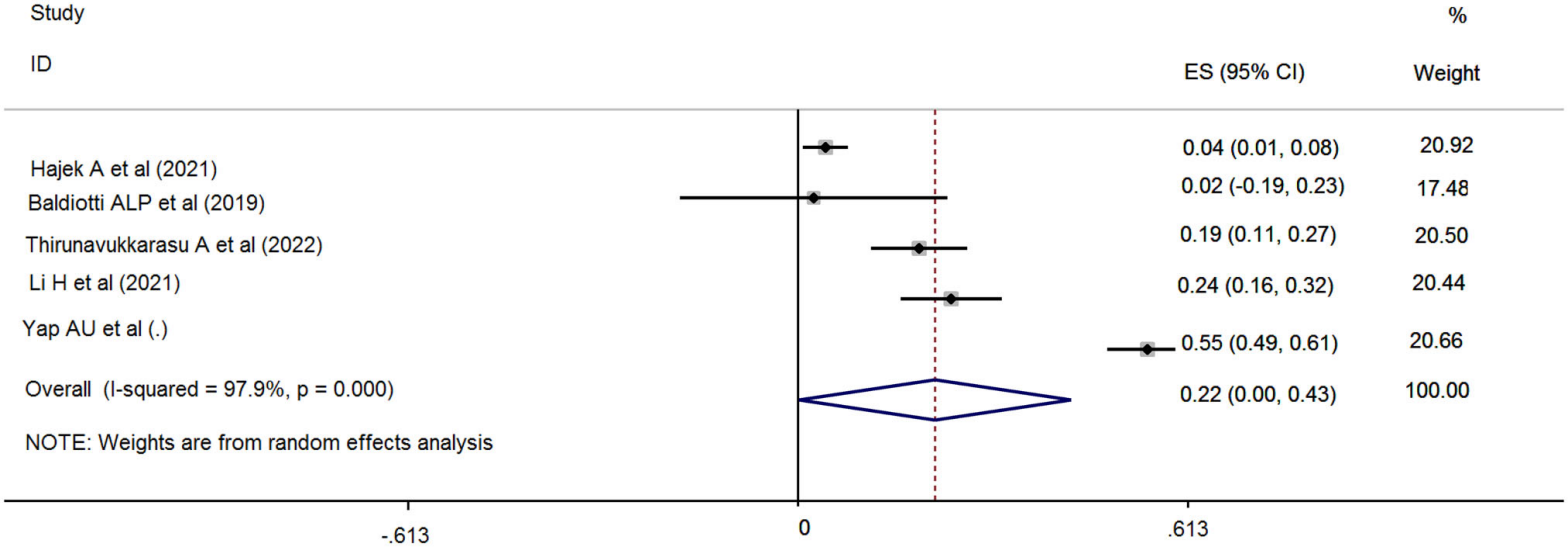
Forest plot displaying the estimated pooled Fishers’ z-score in the association between OHQOL and anxiety.

**Fig. 6:**
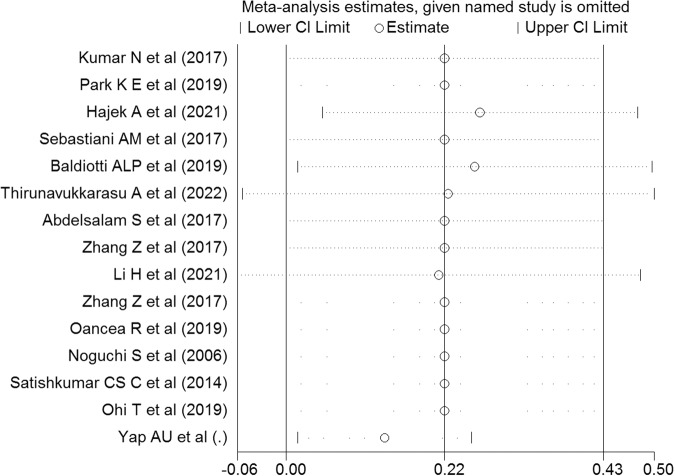
Sensitivity analysis plot assessing the small study effect in the estimated pooled Fishers’ z-score in the association between OHQOL and anxiety.

A weak association between OHQOL and anxiety was suggested by the pooled estimation. Specifically, the Fisher’s z-score was 0.22 with a 95% CI ranging between 0.001 and 0.43 (I^2^ = 97.9%; τ^2^ = 0.06). The forest plot related to the association between OHQOL and anxiety is shown in Fig. 5. Potential publication bias relating to the association between OHQOL and anxiety was not assessed due to the low number of included studies (less than ten studies [28]). Additionally, the sensitivity analysis indicated that no single study impacted the pooled effect size (Fig. 6). According to findings from uni-variable meta-regression, heterogeneity in the association between OHQOL and anxiety was not impacted by any examined variables, including the OHQOL measures. The significant moderators in this association were the target population (explaining 35.68% of the variance, *p* = 0.18), percentage of female participants (explaining 58.07% of the variance, *p* = 0.09), NOS total score (explaining 40.12% of the variance, *p* = 0.16) and OHQOL measure (explaining 58.60% of the variance, *p* = 0.09). Due to the low number of included studies, further multivariable meta-regression analysis was not possible. The identified moderators should be considered in future investigations of associations between OHQOL and anxiety.

## Discussion

With the use of rigorous methods, the present systematic review with meta-analysis obtained the following synthesized results. OHQOL was significant related to depression (Fisher’s z-score of 0.26) and anxiety (Fisher’s z-score of 0.22). The rigorous methodology included the (i) adherence to the PRISMA guidelines [19] with pre-registration [33]; (ii) use of two independent reviewers to screen 5,178 papers; (iii) data collection with a pre-designed Excel sheet to retrieve important information from the included studies; (iv) use of the NOS [22] to evaluate every included study; (v) effect size calculation with the consideration of different forms of numerical findings such as odds ratios and standardized mean differences [23, 24, 33]; (vi) evaluation of the potential heterogeneity severity [26]; (vii) use of Begg’s Tests, Funnel plots, and Jackknife methodologies to consider publication bias [27–29]; and (viii) use of meta-regression [30–32] to identify potential moderator effects in assessing the associations between depression or anxiety and OHQOL.

The association between depression and OHQOL identified in the synthesized data may reflect amotivation regarding oral hygiene behaviors. Individuals with depression often decrease their engagement in daily activities, including self-care and hygiene behaviors [8–12], and a prominent characteristic of depression is amotivation [13]. However, given that studies were cross-sectional in nature, the extent to which poor OHQOL may induce depression or features of depression may promote poor OHQOL warrants additional investigation.

The association between anxiety and OHQOL may also be explained by disrupted oral hygiene behaviors. Given worries, individuals with anxiety may not engage habitually in daily activities, including self-care and hygiene behaviors [8–12].

Given the present findings, several future directions in research and clinical practice may be recommended. First, almost all studies investigating associations between OHQOL and depression and anxiety were conducted in countries with upper intermediate or high-income levels. Therefore, more studies should be conducted in countries with lower intermediate or low-income levels. Second, most included studies in the present systematic review with meta-analysis used self-reported measures to assess OHQOL, depression, and anxiety. Although using self-reports with appropriate psychometric properties is acceptable, measurement biases (e.g., social desirability) exist. Therefore, future studies are needed to use other measures (e.g., measures assessed by healthcare providers) to minimize measurement biases. Third, the significant associations found between the two types of psychological distress (i.e., depression and anxiety) and OHQOL indicate the need for assessing and reducing possible depression or anxiety when healthcare providers design programs targeting oral health improvement.

The present systematic review with meta-analysis has the following limitations. First, all included studies used cross-sectional designs. Therefore, the findings regarding how OHQOL associates with depression and anxiety and OHQOL could not provide insight into causal relationships. In this regard, future longitudinal studies should examine relationships between depression/anxiety and oral health related quality of life. Second, measures of OHQOL in all studies were based on self-reports. Therefore, social desirability biases and recall biases may have influenced findings. Third, no data regarding response rates were described. Thus, the representativeness of the entire sample used for the present systematic review with meta-analysis could be non-representative based on response rates. Fourth, the present systematic review with meta-analysis only included five studies examining associations between OHQOL and anxiety. Therefore, publication bias was not examined for this relationship. Fifth, there were different OHQOL measures used in the synthesized studies. Although the present meta-analysis had used meta-regression model to confirm no/modest/limited impacts of different OHQOL measures on the synthesized findings, future meta-analyses are needed to reevaluate the role of different OHQOL measures when sufficient empirical evidence is reported. Lastly, the present systematic review and meta-analysis included papers with different populations (e.g., older adults, people with diabetes, and the general population). Therefore, the associations between OHQOL and depression/anxiety are likely to be diluted by the features of different populations. For example, the association between OHQOL and depression/anxiety in older adults might not be similar to the association in people with diabetes. In this regard, the associations found in the present study should be interpreted with caution. However, given that the available studies are not sufficient for us to conduct meta-analysis for a specific population, future meta-analyses may need to be conducted when empirical evidence is sufficient for specific populations.

## Conclusion

In conclusion, the synthesized findings from the present systematic review with meta-analysis indicated that both depression and anxiety were weakly associated with individuals’ OHQOL. Although the synthesized associations were not strong, they were significant, indicating that depression and anxiety might also be considered when one wants to improve individuals’ OHQOL. Therefore, when designing programs to improve OHQOL, healthcare providers should consider depression and anxiety. However, given that the present findings were derived from cross-sectional studies, causal relationships cannot be concluded. Additional research is thus needed.

## Data Availability

The data that support the findings of this study are available from the corresponding author, upon reasonable request.
